# CD5L as an Extracellular Vesicle-Derived Biomarker for Liquid Biopsy of Lung Cancer

**DOI:** 10.3390/diagnostics11040620

**Published:** 2021-03-30

**Authors:** Eun-Sook Choi, Hasan Al Faruque, Jung-Hee Kim, Kook Jin Kim, Jin Eun Choi, Bo A. Kim, Bora Kim, Ye Jin Kim, Min Hee Woo, Jae Yong Park, Keun Hur, Mi-Young Lee, Dong Su Kim, Shin Yup Lee, Eunjoo Kim

**Affiliations:** 1 Division of Bi-Fusion Research, Daegu Gyeongbuk Institute of Science and Technology (DGIST), Techno-jungangdaero 333, Dague 42988, Korea; stom96@dgist.ac.kr (E.-S.C.); hasan@dgist.ac.kr (H.A.F.); 2 Division of Electronic Information System Research, Daegu Gyeongbuk Institute of Science and Technology (DGIST), Techno-Jungangdaero 333, Dague 42988, Korea; cell84@dgist.ac.kr; 3 Genomine Research Division, Genomine Inc., Pohang Technopark, Pohang 37668, Korea; gjkim@genomine.com (K.J.K.); boa88@genomine.com (B.A.K.); pupybr@genomine.com (B.K.); yejini1@genomine.com (Y.J.K.); wmh0929@genomine.com (M.H.W.); dskimi@genomine.com (D.S.K.); 4 Department of Biochemistry, School of Medicine, Kyungpook National University, Daegu 41944, Korea; jechoi.9711@gmail.com (J.E.C.); KeunHur@knu.ac.kr (K.H.); 5 Department of Internal Medicine, School of Medicine, Kyungpook National University, Daegu 41944, Korea; jaeyong@knu.ac.kr; 6 Lung Cancer Center, Kyungpook National University Chilgok Hospital, Daegu 41404, Korea; 7 Department of Medical Science, Soonchunhyang University, Asan 31538, Korea; umma7008@gmail.com

**Keywords:** lung cancer, extracellular vesicle, exosome, CD5L, 2-D gel electrophoresis, proteomics, biomarker, liquid biopsy

## Abstract

Cancer screening and diagnosis can be achieved by analyzing specific molecules within serum-derived extracellular vesicles (EVs). This study sought to profile EV-derived proteins to identify potential lung cancer biomarkers. EVs were isolated from 80 serum samples from healthy individuals and cancer patients via polyethylene glycol (PEG)-based precipitation and immunoaffinity separation using antibodies against CD9, CD63, CD81, and EpCAM. Proteomic analysis was performed using 2-D gel electrophoresis and matrix-assisted laser desorption ionization–time-of-flight mass spectrometry (MALDI–TOF MS). The expression of proteins that were differentially upregulated in the EVs or tissue of lung cancer samples was validated by Western blotting. The area under the curve (AUC) was calculated to assess the predictability of each differentially expressed protein (DEP) for lung cancer. A total of 55 upregulated protein spots were selected, seven of which (CD5L, CLEC3B, ITIH4, SERFINF1, SAA4, SERFINC1, and C20ORF3) were found to be expressed at high levels in patient-derived EVs by Western blotting. Meanwhile, only the expression of EV CD5L correlated with that in cancer tissues. CD5L also demonstrated the highest AUC value (0.943) and was found to be the core regulator in a pathway related to cell dysfunction. Cumulatively, these results show that EV-derived CD5L may represent a potential biomarker—detected via a liquid biopsy—for the noninvasive diagnosis of lung cancer.

## 1. Introduction

Lung cancer is the leading cause of cancer-related deaths worldwide, with a five-year survival rate below 21% [1]. It is categorized into small cell lung cancer (SCLC) and non-small cell lung cancer (NSCLC), which constitute 15% and 85% of the lung cancer cases, respectively [2]. Generally, the initial identification of lung cancer can be occurred by X-ray and computed tomography scans (CT), but there can be significant diagnostic errors by missed or wrong diagnosis [3]. Tissue biopsies are then performed to confirm the diagnosis. Liquid biopsy has emerged as an alternative, simple, and less invasive procedure for analyzing disease-specific biomarkers in non-solid biological tissues, including blood, sputum, and saliva [4,5]. In lung cancer, liquid biopsy has been developed as an added tool for screening and early detection of cancer [6]. Hence, this technique has the potential to improve the survival rates of patients with various cancers, including lung cancer. Indeed, detection and surgical dissection of NSCLC, such as adenocarcinoma (AC) and squamous cell carcinoma (SCC), at stage I are associated with favorable five-year survival rates of 70–90% [7].

Liquid biopsy has also been specifically employed for analyzing cancer biomarker expression within extracellular vesicles (EVs) (diameter ~100 nm), which originated by cells for cell-to-cell communication [8]. EVs carry various cargoes, including proteins, miRNAs, mRNAs, and metabolites for intercellular transfer of specific signals. The molecular contents of EVs have been profiled using various methods to identify molecular biomarkers that can reflect the cell status. Next-generation sequencing has been used to analyze the small RNAs and mRNAs present in EVs [9,10]. Moreover, protein profiling is generally performed using one of the two approaches. Top-down proteomics involves the separation of intact proteins without enzymatic treatment and then the proteins are subjected to tandem mass spectroscopy (MS/MS). Alternatively, the bottom-up approach involves the chemical or enzymatic digestion of proteins into smaller peptides, followed by matrix-assisted laser desorption ionization–time-of-flight mass spectroscopy (MALDI–TOF/MS) [11]. The top-down approach provides the advantage of reduced sample complexity, while resolving isoforms, spliced variants, and post-translationally modified (PTM) proteins in a given sample [12]. However, it is limited to identify new biomarkers and not achieved on a large scale due to a lack of intact protein fractionation methods for MS/MS. Bottom-up methods are commonly used for high throughput screening because the separation of peptides is improved compared to that of intact proteins and the sensitivity is higher than the top-down methods. However, the coverage of protein identification from the peptide sequence is limited, and there is an ambiguity of the origin for redundant peptide sequences [13].

In this study, we performed 2-D gel electrophoresis (2-DE)-based protein separation before MALDI–TOF/MS [14], to identify potential EV biomarkers for lung cancer. This bottom-up proteomics was performed to maximize the efficacy of screening for new EV biomarkers of lung cancer. We then selected differentially expressed proteins (DEPs) based on 2-DE gel spot intensity and confirmed their expression levels in patient EVs by Western blotting analysis. Pathway analysis using ingenuity pathway analysis (IPA) software then identified pathophysiological pathways associated with the candidate biomarkers [15]. We also investigated the relationship between the expression of candidate markers in EVs and cancer tissues, and finally proposed an EV biomarker—detectable via a liquid biopsy—that may prove useful for lung cancer screening and diagnosis.

## 2. Materials and Methods

### 2.1. Sample Collection

A total of 60 serum samples (20 serum samples from each AC, SCC, and SCLC patients) from lung cancer patients and 20 normal serum samples were obtained from Kyungpook National University Chilgok Hospital, and the samples were used to identify EV-derived biomarkers for lung cancer. Sample information is listed in Table 1. In addition, 20 pancreatic and colorectal cancer serum samples (10 each) were obtained as references.

### 2.2. Isolation of EVs from Human Sera and Cell Culture Media

EVs were isolated from the serum samples by salting out, followed by magnetic separation. For salting out, 600 μL of serum was centrifuged at 12,000× *g* for 30 min at 4 °C to eliminate cell debris and contaminants. To the supernatant, 25% polyethylene glycol (PEG 2000, Sigma-Aldrich, St. Louis, MO, USA) was added to achieve a final percentage of 12% PEG in serum and incubated on ice for 1 h. The mixture was then centrifuged at 12,000× *g* for 10 min, and precipitation with 12% PEG was repeated twice more. The pellet was resuspended in 200 μL of phosphate-buffered saline (PBS), and 50 μL of microbeads included in the Exosome Isolation Kit Pan for humans and CD326 (EpCAM) MicroBeads for humans (Miltenyi Biotec, Bergisch Gladbach, Germany) were added. The mixture was incubated for 1 h at 25 °C and centrifuged at 12,000× *g* for 30 min to obtain the final EV pellet for proteomic analysis. Two kinds of commercialized kits for EV preparation were purchased from SBI (Cat# EQPL10A-1, Palo Alto, CA, USA) and Invitrogen (Cat# 4478360, Waltham, MA, USA).

BEAS-2B cells (normal lung cells) and A549 (lung carcinoma cells) were cultured in RPMI-1640 culture media with 10% fetal bovine serum (FBS). To isolate EVs from culture media, complete culture media was replaced with 10 mL of EV-free culture media (SBI, Palo Alto, CA, USA) in each 100-pi dish containing 70–80% confluent cells. After 24 h, the culture media from 10 dishes were harvested, and EVs were isolated using the same protocol as that described for enrichment from serum samples.

### 2.3. Characterization of EVs

For transmission electron microscopy (TEM, Tecnai™ G^2^ Spirit, FEI Company, Hillsboro, OR, USA), nanoparticle tracking analysis (NTA, Nanosight NS300, Malvern Instruments, Malvern, UK), and dynamic light scattering (DLS, Zetasizer Nano, Malvern Instruments, Malvern, UK), the final EV pellet was resuspended in 1 mL of PBS. To identify protein biomarkers, Western blotting was performed. Protein extracts were prepared from EVs by lysing them with radioimmunoprecipitation assay (RIPA) buffer (Thermo Fisher Scientific, Waltham, MA, USA) supplemented with a protease inhibitor cocktail (Sigma-Aldrich, St. Louis, MO, USA). Next, the proteins (20 µg) were subjected to Western blotting. Western blotting was performed following standard protocols. Briefly, protein samples were resolved via sodium dodecyl sulfate-polyacrylamide gel electrophoresis (SDS–PAGE) on a 10% gel and electroblotted onto a nitrocellulose membrane using a transfer apparatus (Trans-Blot SD semi-dry transfer cell, BioRad, Hercules, CA, USA), following the manufacturer’s instructions. Antibodies against CD9, CD81, and CD63 were purchased from System Bioscience (SBI, Palo Alto, CA, USA), and antibodies against Calnexin and TSG101 were purchased from Santa Cruz Biotechnology, Inc. (Dallas, TX, USA). All antibodies were raised in mice, and horseradish peroxidase (HRP)-conjugated secondary antibody against mouse IgG (Abcam, Cambridge, MA, USA) was used for colorimetric detection. The enhanced chemiluminescence (ECL) substrate was purchased from Thermo Fisher Scientific (Waltham, MA, USA), and the intensity of ECL was observed using a ChemiDoc™ system (Bio-Rad) under the same condition of ECL development for all samples. The intensity of each band was analyzed using an image analysis software ImageJ ver. 1.51j (http://imagj.net, accessed on 25 May 2020).

### 2.4. Two-Dimensional Gel Electrophoresis of EV-Derived Proteins

All 2-DE reagents, including urea, thiourea, 3-[(3-cholamidopropy) dimethyammonio]-1-propanesulfonate (CHAPS), dithiothreitol (DTT), benzamidine, Bradford solution, acrylamide, iodoacetamide, bis-acrylamide, and sodium dodecyl sulfate (SDS) were purchased from Sigma-Aldrich (ultrapure electrophoresis grade St. Louis, MO, USA). Pharmalyte (pH 3.5–10) was purchased from GE Healthcare (Wauwatosa, WI, USA), and IPG DryStrips (pH 4–10 NL, 24 cm) were purchased from Genomine Inc. (Pohang, Korea).

EV pellets derived from serum samples were resuspended in ice-cold PBS and lysed with 7 M urea, 2 M thiourea containing 4% (*w*/*v*) CHAPS, 1% (*w*/*v*) DTT, 2% (*v*/*v*) pharmalyte, and 1 mM benzamidine. Proteins were then extracted for 1 h at 25 °C by vortexing. After centrifugation at 15,000× *g* for 1 h at 15 °C, the insoluble material was discarded, and the soluble fraction was used for 2-DE. Protein concentration was quantified using the Bradford colorimetric method. IPG strips were equilibrated for 12 h with 7 M urea, 2 M thiourea containing 2% CHAPS, 1% DTT, and 1% pharmalyte with 50 μg of EV-derived proteins. Isoelectric focusing (IEF) was performed using a Multiphor II electrophoresis unit (GE Healthcare, Wauwatosa, WI, USA). The voltage was linearly increased from 150 to 3500 V over a 3 h period for sample entry, followed by a constant 3500 V, with focusing completely after 96 kVh. Prior to 2-DE, the strips were sequentially incubated for 10 min in equilibration buffer (50 mM Tris-Cl, pH 6.8, 6 M urea, 2% SDS, and 30% glycerol), with 1% DTT and 2.5% iodoacetamide. Equilibrated strips were inserted into SDS-polyacrylamide gel electrophoresis (PAGE) gels (20 × 24 cm^2^, 10–16%). SDS–PAGE was performed using the Hoefer DALT 2D system (GE Healthcare, Wauwatosa, WI, USA), as per the manufacturer’s instructions. The 2-DE was performed at 20 °C for 1700 Vh. The gels were then stained with Coomassie Brilliant Blue (Thermo Fisher Scientific, Waltham, MA, USA) as per the manufacturer’s instructions.

### 2.5. Identification of Candidate Biomarkers

Acetonitrile, trifluoroacetic acid (TFA), and α-cyano-4-hydroxycinnamic acid were purchased from Sigma-Aldrich (ultrapure electrophoresis grade St. Louis, MO, USA). Modified porcine trypsin (sequencing grade) was purchased from Promega (Madison, WI, USA).

Quantitative analysis of digitized images was performed using PDQuest (version 8.0, BioRad, Hercules, CA, USA) in accordance with the protocols provided by the manufacturer. The intensity of each spot was normalized to the total valid spot intensity. The spots for DEPs were selected if their expression levels were more than twofold higher than those of normal samples. For protein identification and peptide mass fingerprinting (PMF), protein spots were excised and digested with trypsin. The digested peptide mixture was mixed with α-cyano-4-hydroxycinnamic acid in 50% acetonitrile/0.1% TFA and subjected to MALDI–TOF/MS (Microflex LRF-20, Bruker, Billerica, MA, USA). Spectra were collected from 350 shots per spectrum over the m/z range of 600–3000 and calibrated by two-point internal calibration using trypsin autodigestion peaks (*m*/*z* 842.5099, 2211.1046). The mass list of PMF was analyzed using Mascot (version 2.1, Matrix Science, UK) to search for matching proteins in the NCBI NR database (human; 276,468 entries) [16]. The following parameters were used in the database search: trypsin as the cleaving enzyme, a maximum of one missed cleavage, iodoacetamide as a complete modification of cysteine, oxidation of methionine as a partial modification, monoisotopic masses, and a mass tolerance of 0.1 Da. A minimum score of 48 in Mascot reports was used as the acceptance criterion for PMF. For the identified proteins, receiver operating characteristic (ROC) curve was produced from the spot intensity of each DEP on 2-DE gels using normal and patient samples shown in Table 1, and the area under the curve (AUC) was calculated to determine the predictive power of each DEP as a lung cancer biomarker.

### 2.6. Confirmation of Biomarker Expression in EVs and Tissues

To determine the protein content in EVs and tissues, we performed Western blotting using patient samples listed in Table S1. Nine serum samples (three normal and six patient samples) and 12 tissue samples (six normal and six patient samples) were used for this validation experiment. Proteins derived from EVs and tumor tissues were lysed using RIPA buffer containing protease inhibitor cocktail, and 20 µg of protein was used for SDS–PAGE. The resolved proteins were then transferred to a nitrocellulose membrane using a transblot transfer system (BioRad, Hercules, CA, USA). To detect the target protein of interest, membranes were probed with respective primary antibodies followed by incubation with appropriate horseradish peroxidase-linked secondary antibodies. The following primary antibodies were used: CD5L (LSBio, Seattle, WA, USA), RBP4 (GeneTex, Irvine, CA, USA), CLEC3B, ITIH4, SERFINF1, DBP, and β-actin (Santa Cruz Biotechnology, Inc., Dallas, TX, USA), SAA4, SAA1, and SAA1 C20ORF3 (Abcam, Cambridge, MA, USA), and SERFINC1 and FCN3 (MyBioSource, San Diego, CA, USA). Signals were developed using chemiluminescence Western blotting substrates and ChemiDoc XRS imaging system (BioRad, Hercules, CA, USA). Finally, the relative intensity was quantified using ImageJ (http://imagj.net, accessed on 5 October 2020).

### 2.7. Pathway Analysis of Candidate Biomarkers

For the EV-derived DEPs, pathway analysis was performed using IPA (version 2019, Qiagen, Germantown, MD, USA). The DEPs were uploaded to IPA for core analysis and analyzed with the molecular network in the ingenuity pathway knowledge base (IPKB) [17]. As an outcome of IPA core analysis, functional networks of involved molecules were identified, and the correlations between DEPs and the other molecules in the networks were obtained.

### 2.8. Transfection of Lung Cells with CD5L siRNA

To identify the relationship between CD5L expression in EVs and parent cells, BEAS-2B cells (normal lung cells) and A549 (lung carcinoma cells) were cultured in RPMI-1640 culture media containing 10% fetal bovine serum (FBS). To analyze the mRNA expression of CD5L in parent cells, 2 × 10^5^ cells were seeded per well in six-well plates and incubated overnight at 37 °C with 5% CO_2_. Cells were divided into four groups, three of which were transfected with control siRNA (negative control), siGAPDH (positive control), and siCD5L (target), respectively. The final group was not treated with siRNA (No siRNA control). All siRNAs were purchased from Dharmacon, Inc. (Lafayette, CO, USA); ON-TARGETplus non-targeting pool, ON-TARGETplus glyceraldehyde 3-phosphate dehydrogenase (GAPDH) control pool, and ON-TARGETplus human CD5L siRNA. The transfection of siRNA intoBEAS-2B and A549 cells was performed according to the manufacturer’s instructions. Briefly, a 5 mM solution of siRNA was prepared in an FBS-free RPMI-1640 cell culture medium. Then, in a separate tube, 10 µL of 5 µM siRNA was added to 190 µL of FBS-free medium to prepare a 200 µL siRNA solution. In another tube, 4 µL of DharmaFECT reagent (Lafayette, CO, USA) was added to 196 µL of FBS-free medium to prepare 200 µL of diluted transfection reagent. The contents of both tubes were mixed and incubated for 20 min at 25 °C. Next, 1.6 mL of complete medium containing 10% FBS was added to 2 mL transfection medium, which was then used to replace the culture medium in six-well plates, and incubated for 24 h before mRNA analysis. For protein analysis, the cells were grown in a 100-mm culture dish. A total of 10 milliliters of the transfection medium was prepared per culture dish by adding 50 µL of 5 µM siRNA to 950 µL FBS-free medium, and 20 µL of DharmaFECT reagent to 980 µL of FBS-free medium. Both reagents were added to 8 mL of complete medium, which was used to replace the culture medium in the dish. Protein analysis was performed after 48 h incubation using Western blot analysis as described above.

### 2.9. Analysis of mRNA Expression

Total RNA from BEAS-2B and A549 cells was extracted using the Hybride-R™ RNA Kit (GeneAll^®^™, Seoul, Korea), as per the manufacturer’s instructions. For mRNA analysis, 1 μg of total RNA was reverse transcribed into cDNA using PrimeScript 1st strand cDNA Synthesis Kit (Exiqon, Vedbaek, Denmark). For the determination of mRNA expression, real-time polymerase chain reaction (RT-qPCR) was performed using gene-specific primer pairs (Table S2), and the Roche SYBR-Green^®^ master mix on an ABI7900HT Real-Time PCR System (Thermo Fisher Scientific Waltham, MA, USA). The transcripts were quantified using the following program: 3 min at 95 °C, 35 cycles of 15 s at 95 °C, 25 s at 60 °C, and 25 s at 72 °C. Values for each transcript were normalized to those of GAPDH using the 2^−ΔΔCt^ method [18].

### 2.10. Statistical Analysis

For all experiments, data from three independent experiments were analyzed using Student’s *t*-test and are reported as mean ± SD. Prism 8 (GraphPad Software, Systat Software, San Diego, CA, USA) was used to determine the *p*-values. A *p*-value <0.05 was considered significant.

## 3. Results

### 3.1. Isolation of EVs from Human Serum Samples and Subsequent Characterization

A total of 80 serum samples from lung cancer patients and normal individuals were used for the identification of EV-derived biomarkers, and the patient characteristics are shown in Table 1. The median age at cancer diagnosis was 69 years old (range, 52–83 years old). The median age of normal serum donors was 50 years old (range 47–63 years old). The number of samples in stages I, II, III, and IV were 7, 1, 22, and 30. To exclude biomarkers with reactivity against other cancer types, serum samples from patients with pancreatic cancer and colon cancer (10 samples each) were also analyzed.

**Figure 1:**
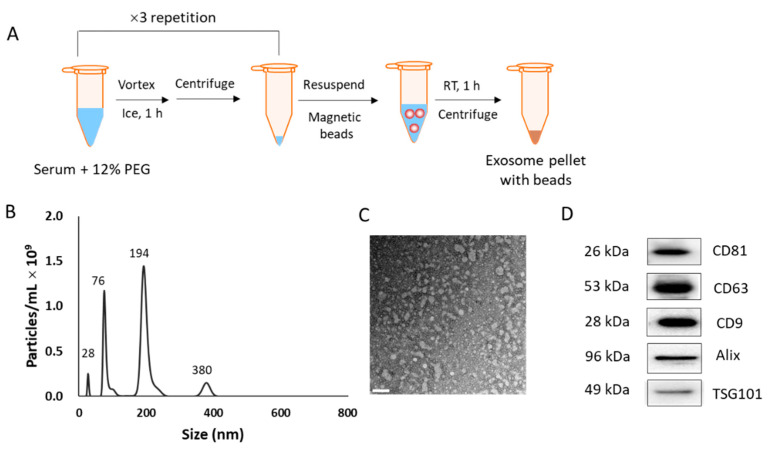
Isolation protocol and characterization of extracellular vesicles (EVs). (**A**) Scheme of the isolation method. (**B**) Nanoparticle tracking analysis of the isolated EVs. (**C**) Transmission electron microscopy image of the isolated EVs. Scale bar = 200 nm. (**D**) Western blotting to check for the presence of EV biomarkers in the EV fraction.

Serum-derived EVs were isolated from the serum (600 μL) of cancer patients and normal individuals, using the method shown in Figure 1A. The final pellet was resuspended in 1 mL of PBS for the characterization of EVs. The size of the resuspended EVs was identified based on the presence of four peaks in NTA (28, 76, 194, and 380 nm; Figure 1B). The TEM image showed clear EV morphology with a size distribution <200 nm (diameter), which supported the NTA-identified sizes (Figure 1C). Since we used four types of antibodies for immunoaffinity-based isolation, the EVs with different sizes were isolated simultaneously. In addition, a peak at 28 nm appears to represent free beads dissociated from EVs [19], while the peak at 380 nm may represent aggregated EV-bead complexes. Although the size distribution was heterogeneous, Western blotting revealed the presence of well-known EV markers, such as CD81, CD63, CD9, Alix, and TSG101 in the isolated EV fraction (Figure 1D). The whole Western blot analyses of these EV biomarkers are provided in Figure S1.

Total proteins from lung cancer patients and normal serum (600 μL) were 813 ± 143 μg and 1438 ± 106 μg, respectively. From pancreatic cancer and colorectal cancer patients, a total of 538 ± 229 μg EV proteins was extracted from the same serum volume.

### 3.2. Protein Profiling for Lung Cancer-Derived EVs

To compare the protein profiles of patient-derived EVs to those of normal EVs, 2-DE was performed. A total of 250 μg of each EV protein was used for 2-DE. As serological proteins are usually co-isolated abundantly with EVs, the number of protein spots in 2-DE could be an indicator of the removal efficiency of serological proteins and the quality of EVs. Figure S2 shows comparative protein spots following PEG precipitation (Figure S2A) and PEG precipitation and magnetic bead separation (Figure S2B). The number of protein spots was extensively decreased by the additional magnetic separation step, which showed the enhanced isolation efficiency of EVs from serum samples. In addition, two more preparation of EVs using commercialized isolation kits produced by SBI (Figure S2C) and Invitrogen (Figure S2D) were performed. The result showed that the preparation of EVs in this study could remove serological proteins efficiently than by two commercialized isolation kits. Taken together with the result of Figure 1D, the EVs were expected to be isolated efficiently using EV-specific antibodies with enhanced purity of them.

Resolution of EV proteins on 2-DE and their analysis, using PDQuest, resulted in the detection of 55 DEP spots, whose expression was found to be increased by over twofold in patient EVs with >95% confidence. All 55 spots were identified using MALDI–TOF/MS. If isotypes or variants of a protein appeared in different spots of a 2-DE gel, we selected a representative isotype with the highest AUC value, using a receiver operating characteristic curve. As a result, the final 22 DEPs with increased fold changes in lung cancer were selected and are presented in Table S3.

Among the 22 DEPs, 10 were selected as potential biomarker candidates (Table 2; Figure S3) based on the following criteria: (1) AUC > 0.750; (2) fold change in pancreatic and colorectal cancer samples < twofold. The AUC of CD5L was found to be 0.943, which was the highest among all candidates.

### 3.3. Confirmation of Biomarker Candidates by Western Blotting

To confirm the increased protein expression detected by MALDI–TOF/MS, the expression of biomarker candidates listed in Table 2 was analyzed by Western blotting. Two serum samples from normal healthy controls and six serum samples from lung cancer patients were used for the isolation of EVs (Table S1).

**Figure 2:**
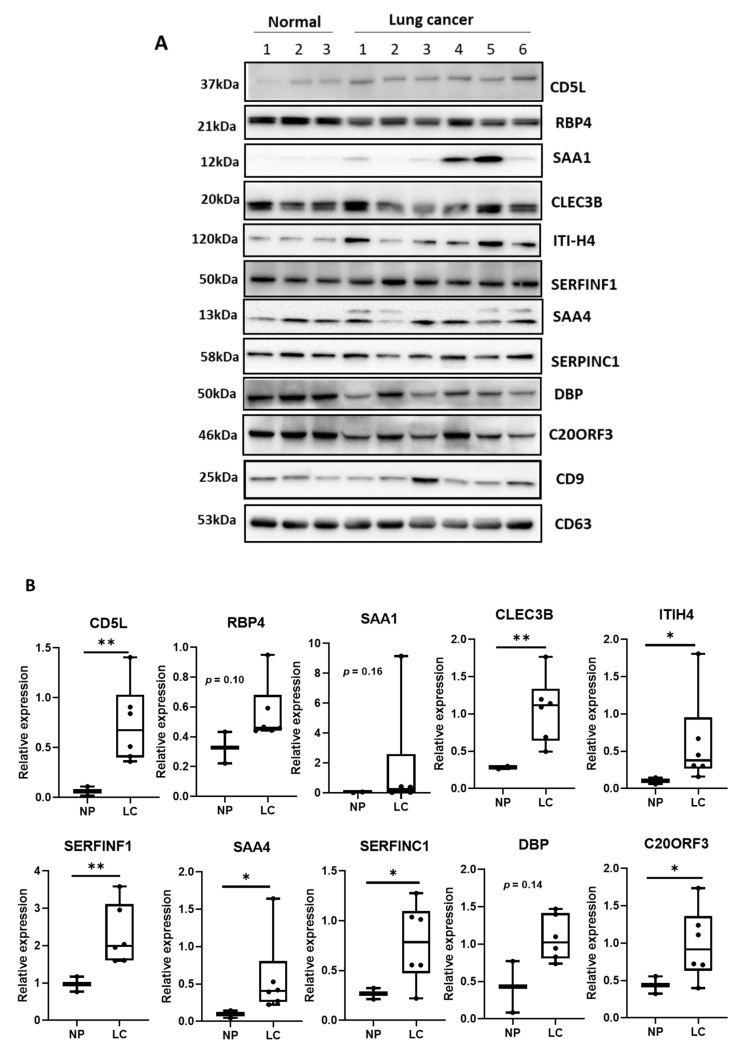
Validation of proteins expressed in EVs derived from patient and normal serum samples. (**A**) Identification of biomarker candidates expressed in serum-derived EVs using Western blotting. SCC, squamous cell carcinoma; AC, adenocarcinoma lung cancer. (**B**) Quantification of protein bands in Western blotting by image analysis. ** *p* < 0.01; * *p* < 0.05. NP, normal person; LC, lung cancer patient.

Figure 2A depicts the proteins expressed in EVs derived from the sera (same volume) of normal and lung cancer patients. The expression of CD5L was enhanced in SCC and AC EVs. Other proteins, such as CLEC3B, ITIH4, SAA1, and C20ORF3 exhibited increased expression in cancer EVs, while RBP4, SAA1, and DBP did not exhibit significant differences between normal and cancer samples. Figure 2B shows the relative expression of each protein compared to that of CD9. Evidently, the levels of CD5L, CLEC3B, and SERFINF1 were significantly increased in cancer EVs (*p* < 0.01), as were those of ITIH4, SAA4, SERFINC1, and C20ORF3 (*p* < 0.05). The expression of RBP4, SAA1, and DBP in cancer EVs was not significantly altered, compared to that in normal EVs. Based on these results, seven proteins, CD5L, CLEC3B, SERFINF1, ITIH4, SAA4, SERFINC1, and C20ORF3 (AMAP) were confirmed as being upregulated in EVs and were therefore considered as potential lung cancer biomarkers.

### 3.4. Expression of EV Proteins in Cancer Tissues

**Figure 3:**
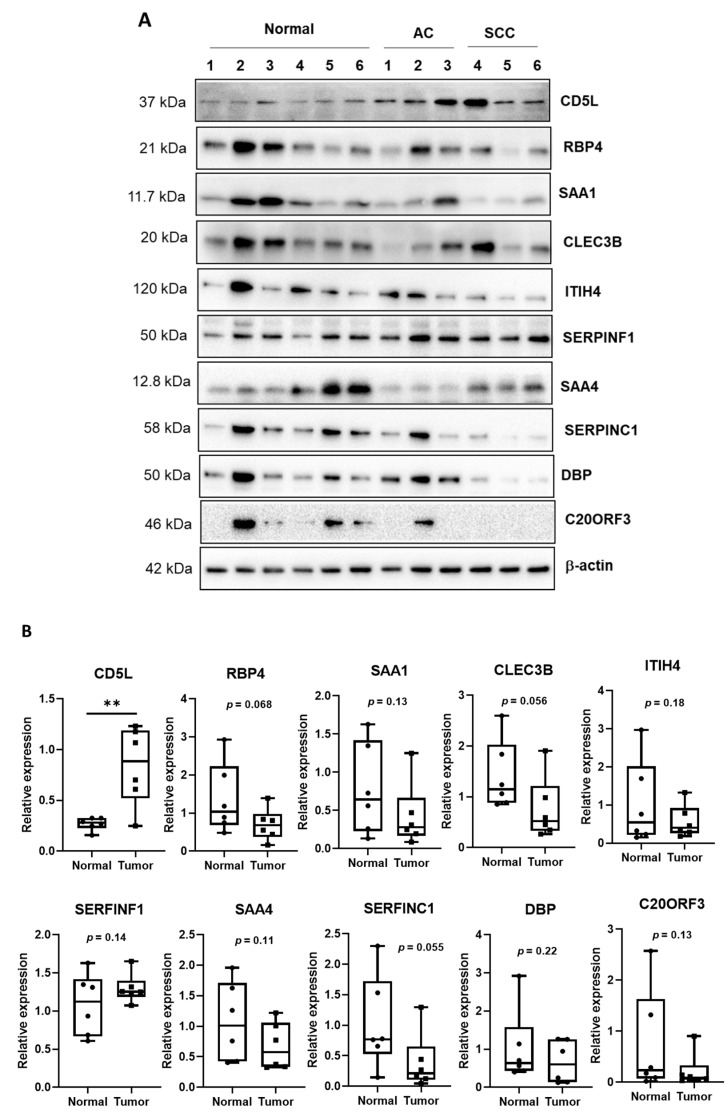
Expression of candidate biomarkers in normal and cancer tissues. (**A**) Western blotting to check for the expression of candidate biomarkers in normal and cancer tissues. The normal tissues were obtained by pairing with cancer tissues from a patient. AC, adenocarcinoma lung cancer; SCC, squamous cell carcinoma. (**B**) Quantitative analysis of Western blot analysis. ** *p* < 0.01.

The expression levels of ten biomarker candidates in cancer tissues were analyzed by Western blotting. A total of 12 tissues, including normal and cancer tissues, were used (Table S1). The expression of CD5L was enhanced in both AC and SCC tissues and in the EV fraction (Figure 3A). However, the other proteins were not observed to be upregulated in cancer tissues. The relative amount compared to β-actin was calculated and is shown in Figure 3B. The expression of only CD5L was significantly upregulated in cancer tissue compared to that in normal tissues (*p* < 0.01); the expression of all other proteins decreased or proteins were expressed at similar levels in cancer tissues and normal tissues, without any significant difference. We, therefore, proposed that CD5L represents the key EV molecule among the biomarker candidates, the expression of which corresponds with lung cancer and may represent an effective biomarker in liquid biopsies.

### 3.5. Biological Networks Related to the Biomarkers

**Figure 4:**
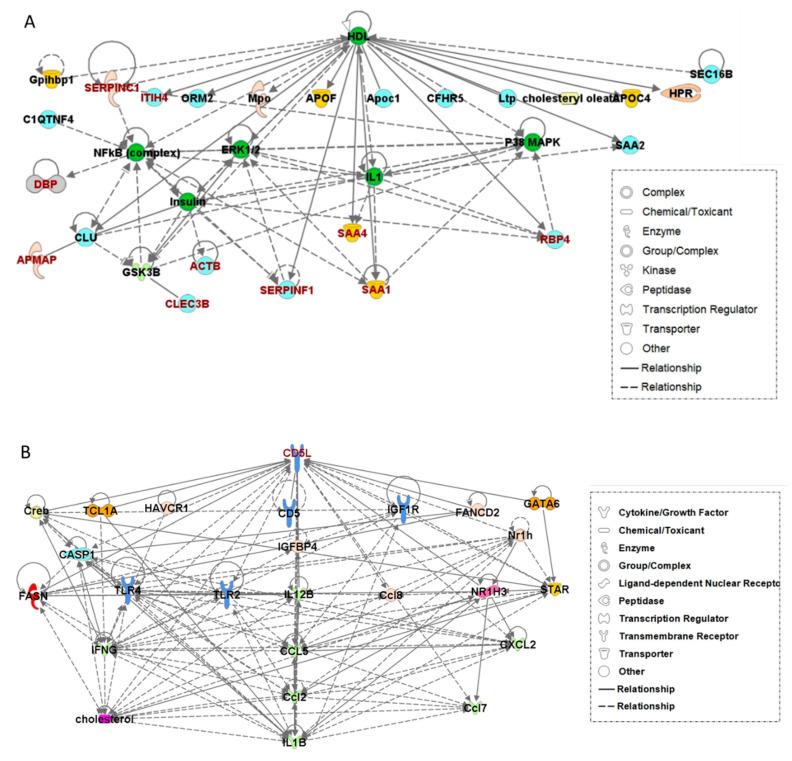
Network analysis performed using ingenuity pathway analysis (IPA). (**A**) Network 1 including nine of the previously identified differentially expressed proteins. (**B**) Network 2 with CD5L as the hub protein.

The 22 DEPs listed in Table S3 were analyzed for their involvement in functional biological networks using the IPA database (Table S4). The top functions of Network 1, comprising 10 proteins (C20ORF3 (AMAP), CLEC3B, DBP, ITIH4, RBP4, SAA1, SAA4, SERFINC1, ACTB, and SERFINF1), were categorized into the following: lipid metabolism, molecular transport, and small-molecule biochemistry (Figure 4A). Among these 10 DEPs, all (except ACTB) were included in the biomarker candidates listed in Table 2. Meanwhile, CD5L was located within Network 2, which was related to infectious disease, inflammatory disease, organismal injury, and abnormalities (Figure 4B).

The molecular correlation in Network 1 shows that the nine candidate biomarkers proposed in this study were directly or indirectly related to each other and also associated with high-density lipoprotein (HDL) regulation. Meanwhile, Network 2 contained CD5L as the hub protein, i.e., ranked the highest in the regulatory hierarchy. This result may support the importance of CD5L as an EV-derived biomarker.

### 3.6. Correlation of CD5L Level in EVs and Cancer Cells

The CD5L levels in patient serum-derived EVs may be related to its expression in lung cancer tissues. To investigate this potential association, CD5L expression in lung cell lines (normal lung BEAS-2B cells and lung cancer A549 cells) was inhibited using siCD5L. EVs were then isolated from the culture media of the transfected cells and changes in cellular and EV CD5L expression were assessed.

**Figure 5:**
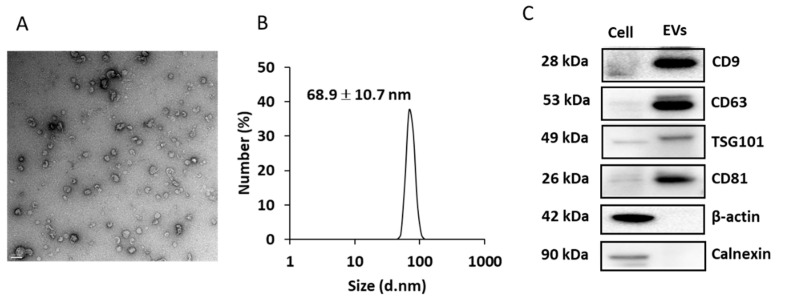
Characterization of the EVs isolated from culture media. (**A**) EVs imaged using transmission electron microscopy (TEM). Scale bar = 100 nm. (**B**) dynamic light scattering (DLS) analysis of the isolated EVs. (**C**) EV markers analyzed by Western blotting.

Figure 5A shows the morphology of EVs secreted into the culture media. The particle size was determined to be 68.9 ± 10.7 nm by DLS (Figure 5B). EV markers, such as CD9, CD63, TSG101, and CD81 were enriched in the EV fraction (compared to that in cell extracts; Figure 5C). Beta-actin and calnexin were used as cellular biomarkers, which were expressed in cell lysate but not appeared in EV fractions (Figure 5C).

**Figure 6:**
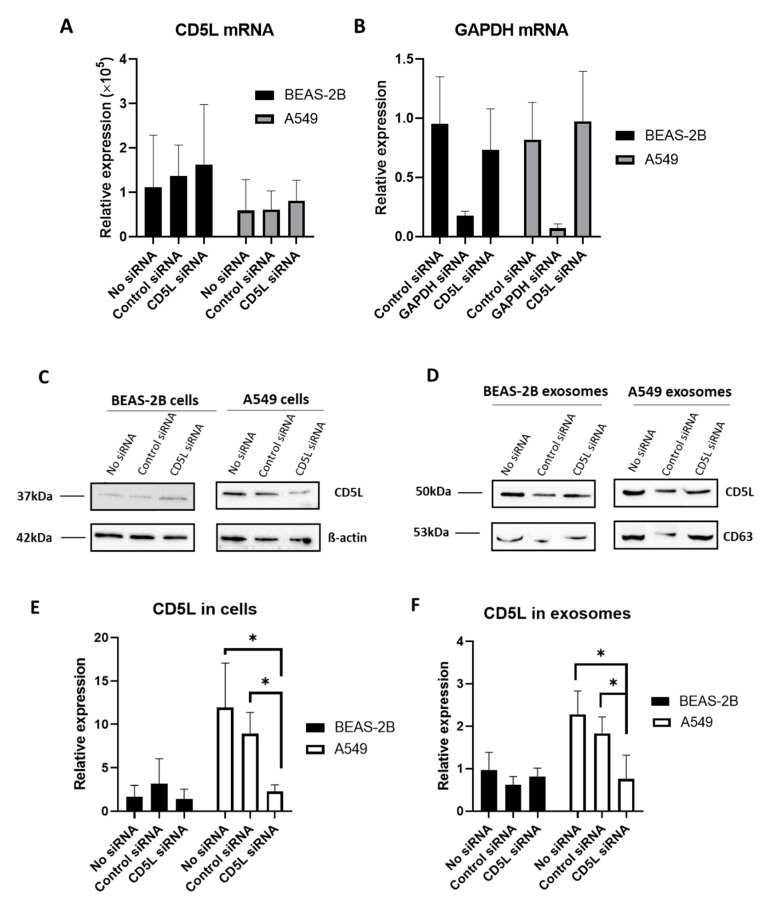
Transfection of siCD5L into BEAS-2B and A549 cells. (**A**) CD5L mRNA expression analyzed by RT-qPCR. (**B**) Glyceraldehyde 3-phosphate dehydrogenase (GAPDH) mRNA expression analyzed by RT-qPCR. (**C**) CD5L protein expression in parent cells analyzed by Western blotting. (**D**) CD5L protein expression in EVs analyzed by Western blotting. (**E**) Quantitative analysis of CD5L by Western blotting in cells. (**F**) Quantitative analysis of CD5L by Western blotting in EVs. No siRNA, control group without transfection of siRNA; Control siRNA, cells transfected with control siRNA as negative control; GAPDH siRNA, cells transfected with GAPDH siRNA as positive control; CD5L siRNA, cells transfected with CD5L siRNA. * *p* < 0.05.

The mRNA expression of CD5L was not altered upon transfection of BEAS-2B and A549 cells with either siCD5L or siGAPDH; however, a marked decrease in the expression of GAPDH mRNA was observed upon transfecting these cells with siGAPDH (Figure 6B).

In contrast, Western blotting revealed that the expression of CD5L was altered upon transfection with siCD5L. Specifically, CD5L expression in A549 cells, following transfection with siCD5L, was significantly decreased, compared to that in untreated and control siRNA-transfected cells (Figure 6C,E). Additionally, the Human Protein Atlas database (http:///www.proteinatlas.org, accessed on 14 October 2020) [20] did not reveal the presence of CD5L mRNA in A549 cells, while the presence of CD5L protein was reported in lung tissues based on histological analyses. In line with this, in the current study, CD5L mRNA was found to be expressed at very low levels in BEAS-2B and A549 cells in all groups, including the nontreated control; however, CD5L protein was found to be expressed at high levels in A549 cells, compared to that in BEAS-2B cells, as determined by Western blotting.

To investigate whether the downregulation of CD5L following transfection with siCD5L altered the CD5L levels in the EV fraction, EVs were isolated from cells transfected with siCD5L, and Western blotting was performed. After quantification and normalization of the band intensity of CD5L to that of CD63, the relative expression of CD5L in EVs was found to be significantly decreased in the siCD5L-transfected A549 cells (*p*-value = 0.045; Figure 6D,F).

## 4. Discussion

The identification of EV biomarkers may differ depending on the isolation method used as each strategy has inherent advantages and disadvantages and provides a differential yield of EVs based on the parent cells. The most common EV isolation methods include ultracentrifugation, ultrafiltration, polymer-based precipitation, immunoaffinity capture, and size exclusion chromatography. In this study, we developed a two-step protocol, polymer-based precipitation using PEG and immunoaffinity separation based on the use of magnetic beads. We found that the use of 12% PEG solution maximized the recovery of vesicular materials, while immunoaffinity separation using CD9, CD63, CD81, and EpCAM antibody-immobilized magnetic beads was performed for the specialized separation of EVs. Using this protocol, 500–1000 µg of protein was isolated from 600 µL serum, which was sufficient for use in the downstream 2-DE analysis.

Considering that the primary aim of this study was to identify new biomarkers for the diagnosis of lung cancer, it was considered more important to collect cancer-specific EVs than to achieve homogeneity in terms of EV size and morphology. Figure 1B,C shows that our protocol resulted in the co-isolation of large (194 nm) and small (76 nm) EVs, with a significant proportion of the vesicles <200 nm in diameter. As shown in Figure 1D, EV-specific biomarkers, such as CD81, CD63, CD9, Alix, and TSG101 were clearly identified, indicating that the method was efficient for enriching EVs from serum samples. These markers were provided as examples of proteins commonly found in mammalian cell-derived EVs [21]. Among them, CD63 was reported to be present in EVs with a broad size range (<50 nm to >200 nm) [22]. Moreover, EpCAM is known to be expressed in several types of cancer-cell-derived EVs [23,24] and is also reported to be a prognostic biomarker in NSCLC [25]. Hence, the introduction of these four antibodies for immunoaffinity separation was expected to enrich the EVs specific to lung cancer in the circulation system, despite the heterogeneous size distribution. From the EV extract, prepared in this study, 55 DEPs were selected by enhanced spot density, accounting for a larger DEP pool compared to that of previous studies, such as in cases of rheumatoid arthritis and liver cancer detection (28 and 24 DEPs, respectively) [26,27]. In addition, all DEPs were categorized as EV-associated proteins by the Exocarta database (http://www.exocarta.org, accessed on 8 February 2020).

Two criteria were employed for the selection of biomarker candidates from DEPs. The first was that the AUC value was >0.750 in lung cancer diagnosis, while the second criterion included lung cancer-specific biomarkers by excluding proteins whose expression was increased by more than twofold in pancreatic or colorectal cancer. This led to the selection of 10 proteins as candidates. Quantitative analysis of the expression of these candidates by Western blotting confirmed that seven candidates, including CD5L, were significantly upregulated in EVs. However, in cancer tissues, the expression of only CD5L was found to be increased; hence, the EV level of other candidates was not related to the expression in cancer tissues. This result indicated that CLEC3B, ITIH4, SERFINF1, SAA4, SERFINC1, and C20ORF3 cannot serve as effective biomarkers for lung cancer. CD5L was also detected in the EVs from bronchoalveolar lavage of lung cancer patients, but there was no correlation to the tumor progress [28]. It is attributed to the different sample numbers (*n* = 24 for bronchoalveolar lavage) to our study (*n* = 80), the different isolation methods used in the two studies (ultracentrifugation vs. magnetic separation), and the difference in the sample origin.

The cargo of cancer-derived EVs reportedly contributes to cancer development. For example, cancer-derived EVs regulate endothelial angiogenic responses by promoting the formation of the endothelial tubule networks, which could lead to cancer malignancy [29]. Hence, the protein content in EVs is reflective of their origin and varies depending on the type of host cells. Proteins are generally loaded into EVs with the help of the ESCRT complex; therefore, dissection of the ESCRT-dependent pathway is necessary for elucidating the specific functions in the selective import of proteins into EVs [30]. Although the mechanisms underlying EV biogenesis have been previously described, the sorting of cellular proteins into EVs has not been explored well [31]. Recent studies have revealed that the protein levels in EVs do not always reflect their levels in parent cells. For example, CD82 is not expressed in lymphoid tumor cells but is overexpressed in the EVs derived from these very same lymphoid cells [32]. Upon comparing the proteome profiles of EVs from three types of pancreatic cancer cells, it was found that pancreatic ductal adenocarcinoma (PDAC)-cell-derived EVs share less than 50% of their proteins with the parent cells [33]. Hence, some EV-derived proteins from lung cancer may not have originated from the tumor itself but may rather be induced during tumor progression via the circulatory system.

To investigate the molecular function of biomarkers, including CD5L, IPA was performed for the DEPs listed in Table S3. Two separate networks were proposed; Network 1 contained 10 DEPs including nine candidate biomarkers, while CD5L served as the hub protein in Network 2. Results show that 10 molecules were either directly or indirectly associated with each other, while HDL represented the hub molecule in Network 1. Several DEPs, such as APOE, APOA1, and APOL1, are known as representative HDL-associated molecules; however, they were excluded as biomarker candidates due to high fold changes observed in pancreatic or colorectal cancers. Therefore, HDL may be co-isolated with EVs, resulting in certain detected DEPs actually being HDL-associated molecules. In fact, previous studies have reported that HDL can become co-isolated with EVs due to their similar density (1.063–1.21 g/mL) [34]. Among the candidate biomarkers in Network 1, SAA1, SAA4, ITIH4, SERFINF1, and SERFINC1 have been reported to be closely associated with lipid metabolism [35,36,37,38], while RBP4 and DBP are associated with molecular transport [39,40], and CLEC3B is involved in the regulation of HDL-related diseases [41]. Furthermore, SAA, ITIH4, and C20ORF3 (APMAP) have recently been associated with HDL in acute phase response platelet activation [42]. However, the contamination of HDL appeared to be minimized because we used immunoaffinity separation during the isolation of EVs, not ultracentrifugation or ultrafiltration. Moreover, APOE, a representative component of HDL, is also released via EVs from cancer and neuronal cells [43,44]. Therefore, the DEPs in Network 1 could be considered as EV-derived molecules, with functions closely related to HDL and lipid metabolism, which are processes that are intimately related to lung carcinogenesis.

In Network 2, CD5L was found to be a regulatory molecule and a potential EV biomarker for liquid biopsy due to its synchronized expression in cancer tissues and EVs of lung cancer samples. CD5L is also known as apoptosis inhibitor 6 (API6) and has been reported to inhibit the activity of fatty acid synthase, in addition to inducing lipolysis during the progression of obesity [45]. Moreover, it promotes lung tumorigenesis by blocking the mechanism of lung epithelial apoptosis that would normally support immunosurveillance [46].

To verify the association between CD5L expression levels in EVs and parent cells in lung cancer, the expression of CD5L in parent A549 cells was blocked using siCD5L, following which its expression in the released EVs was analyzed. Altered mRNA expression was not observed in A549 or BEAS-2B cells; however, decreased CD5L protein expression was apparent in A549 cells following transfection with siCD5L. The levels of CD5L protein in EVs were also significantly decreased with respect to those in the control groups, i.e., untreated and control siRNA-transfected cells (*p* = 0.045 and 0.011, respectively). This result indicated that the elevated levels of CD5L in patient EVs could be related to the elevated expression in cancer tissues. However, the fact that CD5L is also expressed in macrophages, which induce local and systemic inflammation [47], could provide diverse perspectives regarding the origin and regulatory mechanism of EV CD5L in lung cancer. For example, it is reported that CD5L expression was controlled by LXR/RXR promotors [15], which was activated in an oncogenic state to induce the expression of CD5L. This could be related to the sole inhibitory effect for CD5L expression by siCD5L in A549 cells, not by BEAS-2B cells, as shown in Figure 6C,E.

## 5. Conclusions

Cumulatively, the results suggest that CD5L can be used as a lung cancer-specific EV biomarker for liquid biopsy because its expression is linked to cancer origin, and it represents a core regulatory molecule with respect to functions associated with lung cancer. In addition, CLEC3B, ITIH4, SERFINF1, SAA4, SERFINC1, and C20ORF3, whose expression was significantly increased in EVs derived from lung cancer patients, can be included as potential biomarkers. These proposed biomarkers were functionally related to lung cancer progression via lipid and cholesterol metabolism and could be considered as targets for cancer therapeutics. Moreover, the isolation of EVs from human serum and EV protein profiling could facilitate the use of liquid biopsy for predicting lung cancer progression by measuring CD5L expression in circulating EVs.

## Figures and Tables

**Table 1:** Serum samples used for proteomic screening of biomarkers.

Disease Type	No. of Samples	Age (Mean, Range)	Sex (Male/Female)	Number of Samples (Stage I, II, III, IV)
Normal	20	50 (47–63)	7/13	-
Lung cancer				
AC	20	65 (53–81)	10/10	6, 0, 5, 9
SCC	20	72 (52–83)	16/4	1, 0, 11, 8
SCLC	20	70 (52–82)	18/2	0, 1, 6, 13
Total	60	69 (52–83)	44/16	7, 1, 22, 30

Abbreviations: AC, adenocarcinoma; SCC, squamous cell carcinoma; SCLC, small cell lung cancer.

**Table 2:** Properties of top 10 candidate lung cancer biomarkers selected from the list of differentially upregulated proteins obtained using matrix-assisted laser desorption ionization–time-of-flight mass spectrometry (MALDI–TOF MS).

Protein	Symbol	AUC	Sensitivity	Specificity	SCLC *	AC *	SCC *	PanC *	CRC *
CD5 antigen-like	CD5L	0.943	92.9	94.1	4.4	4.1	4.0	0.3	0.4
Retinol binding protein 4	RBP4	0.917	90.5	88.2	13.0	22.8	18.7	0.1	0.1
Serum amyloid A beta	SAA1	0.893	78.6	100.0	18.3	115.0	168.5	1.0	1.0
Tetranectin	CLEC3B	0.887	88.1	76.5	3.3	16.7	9.0	0.0	0.0
Inter-alpha (globulin) inhibitor H4	ITIH4	0.873	81.0	88.2	9.2	7.5	4.8	0.0	0.0
Serpin peptidase inhibitor, clade F	SERPINF1	0.833	83.3	76.5	2.1	2.5	2.3	0.5	0.5
Serum amyloid A-4	SAA4	0.833	66.7	100.0	22.2	8.7	29.2	1.0	1.0
Serpin peptidase inhibitor, clade C	SERPINC1	0.824	71.4	88.2	10.2	10.8	15.4	0.5	0.5
Vitamin D binding protein	DBP	0.798	59.5	100.0	131.5	136.0	79.9	1.0	1.0
Chromosome 20 open reading frame 3	C20ORF3 (APMAP)	0.753	73.8	82.4	2.9	2.8	2.5	0.0	0.0

*: SCLC, small cell lung cancer; AC, adenocarcinoma; SCC, squamous cell carcinoma; PanC, pancreatic cancer; CRC, colorectal cancer.

## Data Availability

The EV-originated proteomic data are available in supplementary material, Table S3.

## Supplementary Materials

The following are available online at https://www.mdpi.com/article/10.3390/diagnostics11040620/s1, Table S1: Serum and tissue samples used for the validation of the biomarker candidate, Table S2: Gene-specific primers used in this study, Table S3: List of selected DEPs by MALD–TOF/MS, Table S4: Two networks identified by IPA network analysis, Figure S1: Zoomed protein spots in 2-D gel electrophoresis, Figure S2. Images for 2-DE of EVs isolated by (A) PEG precipitation, (B) PEG precipitation and magnetic bead-based isolation, (C) SBI exosome isolation kit, and (D) Invitrogen exosome isolation kit, Figure S3. Zoomed protein spots in 2-D gel electrophoresis following Coomassie Brilliant staining, using serum-originated exosomes.
