# Supplementary material for: CD5L as an Extracellular Vesicle-Derived Biomarker for Liquid Biopsy of Lung Cancer
Source: Diagnostics (Basel). 2021 Mar 30;11(4):620. doi: 10.3390/diagnostics11040620 (PMC8067192; doi:10.3390/diagnostics11040620)
Supplement: Supplementary file 1 [file diagnostics-11-00620-s001.pdf]

Supporting Information

CD5L as an Extracellular Vesicle-Derived Biomarker for Liquid Biopsy of Lung Cancer

Eun-Sook Choi [1,†], Hasan Al Faruque [1,†], Jung-Hee Kim [2], Kook Jin Kim [3], Jin Eun Choi [4], Bo A Kim [3], Bora Kim [3], Ye Jin Kim [3], Min Hee Woo [3], Jae Yong Park [5,6], Keun Hur [4], Mi-Young Lee [7], Dong Su Kim [3], Shin Yup Lee [5,6,*] and Eunjoo Kim [2,*]

[1] Division of Bi-Fusion Research, Daegu Gyeongbuk Institute of Science and Technology (DGIST), Techno-jungangdaero 333, Dague 42988, Korea; stom96@dgist.ac.kr (E.-S.C.); hasan@dgist.ac.kr (H.A.F.).

[2] Division of Electronic Information System Research, Daegu Gyeongbuk Institute of Science and Technology (DGIST), Techno-jungangdaero 333, Dague 42988, Korea; cell84@dgist.ac.kr

[3] Genomine Research Division, Genomine Inc., Pohang Technopark, Pohang 37668, Korea; gjkim@genomine.com (K.J.K.); boa88@genomine.com (B.A.K.); pupybr@genomine.com (B.K.); yejini1@genomine.com (Y.J.K.); wmh0929@genomine.com (M.H.W.); dskimi@genomine.com (D.S.K.)

[4] Department of Biochemistry, School of Medicine, Kyungpook National University, Daegu 41944, Korea; jechoi.9711@gmail.com (J.E.C.); KeunHur@knu.ac.kr (K.H.)

[5] Department of Internal Medicine, School of Medicine, Kyungpook National University, Daegu 41944, Korea; jaeyong@knu.ac.kr

[6] Lung Cancer Center, Kyungpook National University Chilgok Hospital, Daegu 41404, Korea

[7] Department of Medical Science, Soonchunhyang University, Asan 31538, Korea; umma7008@gmail.com

* Correspondence: shinyup@knu.ac.kr (S.Y.L.); ejkim@dgist.ac.kr (E.K.); Tel: +82-53-200-2632 (S.L.); Tel.: +82-53-785-2530 (E.K.)

† These authors contributed equally to this work.

Table 1. Serum and tissue samples used for the validation of the candidate biomarkers.

Sample type	Disease	Number	Age (mean, range)	Gender (male/female)
Serum	Normal	3	43 (26–58)	1/2
	Lung cancer	6	72 (62–76)	6/0
Tissue	Normal	6	69 (58–86)	6/0
	SCC	3	73 (65–86)	3/0
	AC	3	65 (58–72)	3/0

Table S2. Gene-specific primers used in this study.

Gene	Sense	Antisense
CD5L	AAGGGACGCGTGGAAGTG	GCCTGTCTGGCACACGGTAT
GAPDH	GGCATCCTGGGCTACACTGA	GAGTGGGTGTCGCTGTTGAA

Table S3. List of selected DEPs by MALD-TOF/MS, including a representative isotype or a variant for a protein. Proteins written in bold are the biomarker candidates satisfying two criteria.

No.	Protein Name	Symbol	AUC	Sens	Spec	Fold Change				
						SCLC	ADC	SCC	Pan	Col
**1**	**CD5 antigen-like**	**CD5L**	**0.943**	**92.9**	**94.1**	**4.4**	**4.1**	**4.0**	**0.3**	**0.4**
2	**Retinol-binding protein 4**	**RBP4**	**0.917**	**90.5**	**88.2**	**13.0**	**22.8**	**18.7**	**0.1**	**0.1**
3	**Serum amyloid A beta**	**SAA1**	**0.893**	**78.6**	**100.0**	**18.3**	**115.0**	**168.5**	**1.0**	**1.0**
4	**Tetranectin**	**CLEC3B**	**0.887**	**88.1**	**76.5**	**3.3**	**16.7**	**9.0**	**0.0**	**0.0**
5	Muscle clathrin heavy chain	CLTC	0.887	90.5	76.5	4.8	5.4	5.9	3.2	4.9
**6**	**Inter-alpha (globulin) inhibitor**	**ITIH4**	**0.873**	**81.0**	**88.2**	**9.2**	**7.5**	**4.8**	**0.0**	**0.0**
7	Apolipoprotein E	APOE	0.854	97.6	76.5	3.3	3.9	4.6	2.7	0.0
8	**Serpin peptidase inhibitor, clade F**	**SERPINF1**	**0.833**	**83.3**	**76.5**	**2.1**	**2.5**	**2.3**	**0.5**	**0.5**
9	**Serum amyloid A-4**	**SAA4**	**0.833**	**66.7**	**100.0**	**22.2**	**8.7**	**29.2**	**1.0**	**1.0**
10	Haptoglobin	HP	0.828	76.2	94.1	8.3	13.6	9.4	0.7	4.8
11	**Serpin peptidase inhibitor, clade C**	**SERPINC1**	**0.824**	**71.4**	**88.2**	**10.2**	**10.8**	**15.4**	**0.5**	**0.5**
12	Mannan-binding lectin serine protease 2	MASP2	0.823	78.6	82.4	1.7	18.7	14.0	0.4	3.0
**13**	**Vitamin D-binding protein**	**DBP**	**0.798**	**59.5**	**100.0**	**131.5**	**136.0**	**79.9**	**1.0**	**1.0**
14	Beta actin	ACTB	0.793	88.1	64.7	3.0	3.3	2.9	0.2	0.8
15	Apolipoprotein A1	APOA1	0.787	66.7	94.1	0.8	14.6	6.9	42.5	27.8
16	Ficolin-3	FCN3	0.782	78.6	76.5	2.5	2.7	5.7	1.9	0.0
17	Complement C4-B	C4B	0.779	73.8	82.4	2.2	0.6	13.7	0.2	5.9
18	Haptoglobin alpha 2	HP	0.776	66.7	82.4	1.4	8.5	4.2	0.6	1.3

**19**	**Chromosome 20 open reading frame 3**	**C20ORF3 (APMAP)**	**0.753**	**73.8**	**82.4**	**2.9**	**2.8**	**2.5**	**0.0**	**0.0**
20	Complement 9	C9	0.731	54.8	100.0	19.6	16.6	6.6	0.1	0.1
21	Apolipoprotein L1	APOL1	0.686	76.2	70.6	1.0	2.3	1.6	0.0	0.0
22	Transthyretin chain A	TTR1	0.681	59.5	82.4	11.8	2.7	6.1	0.0	0.0

Table S4. Two networks identified by IPA network analysis, using selected DEPs in Table S2.

ID	Top Diseases and Functions	Molecules in Network	Score	Focus Molecule
1	Lipid metabolism, Molecular transport, Small molecule biochemistry	ACTB, alcohol, **APMAP**, APOC1, APOC4, APOF, BCP crystal, C1QTNF4, CFHR5, cholesteryl oleate, **CLEC3B**, CLU, **DBP**, ERK1/2, Gpihbp1, GSK3B, HDL, HPR, hyodeoxycholic acid, IL1, Insulin, **ITIH4**, LTP, MPO, NFkB, ORM2, P38 MAPK, **RBP4**, **SAA1**, SAA2, **SAA4**, SEC16B, selenodiglutathione, **SERPINC1**, **SERPINF1**	28	9
2	Infectious diseases, Inflammatory disease, Organismal injury and abnormalities	13-hydroxyoctadecadienoic acid, 15-hydroxyeicosapentaenoic acid, 16-hydroxy-docosahexaenoic acid, 17-hydroxydocosahexaenoic acid, 20-hydroxydocosahexaenoic acid, 24(S),25-epoxycholesterol, 8-hydroxydocosahexaenoic acid, 8-hydroxyeicosatetraenoic acid, 9-hydroxyoctadecadienoic acid, 9S-hydroxy-10E, 12Z, 15Z-octadecatrienoic acid, CASP1, Ccl2, CCL5, CCL7, CCL8, CD5, **CD5L**, cholesterol, CREB, CXCL2, FANCD2, FASN, GATA6, HAVCR1, IFNG, IGF1R, IGFBP4, IL12B, IL1B, NR1H, NR1H3, STAR, TCL1A, TLR2, TLR4	2	1

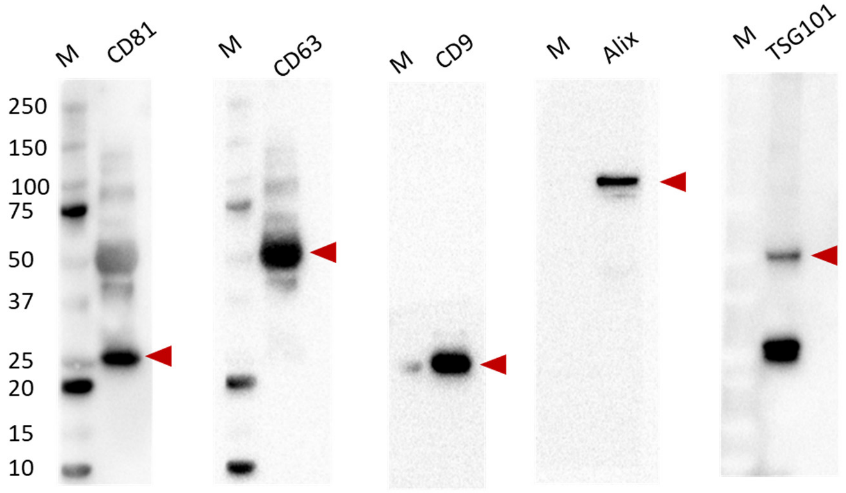

Figure S1. Western blotting analysis of EV biomarkers, such as CD81, CD63, CD9, Alix and TSG101. Whole blot of proteins from SDS-PEGE of exosomes isolated form a patient serum, and cropped images of each biomarker are provided in Figure 1D.

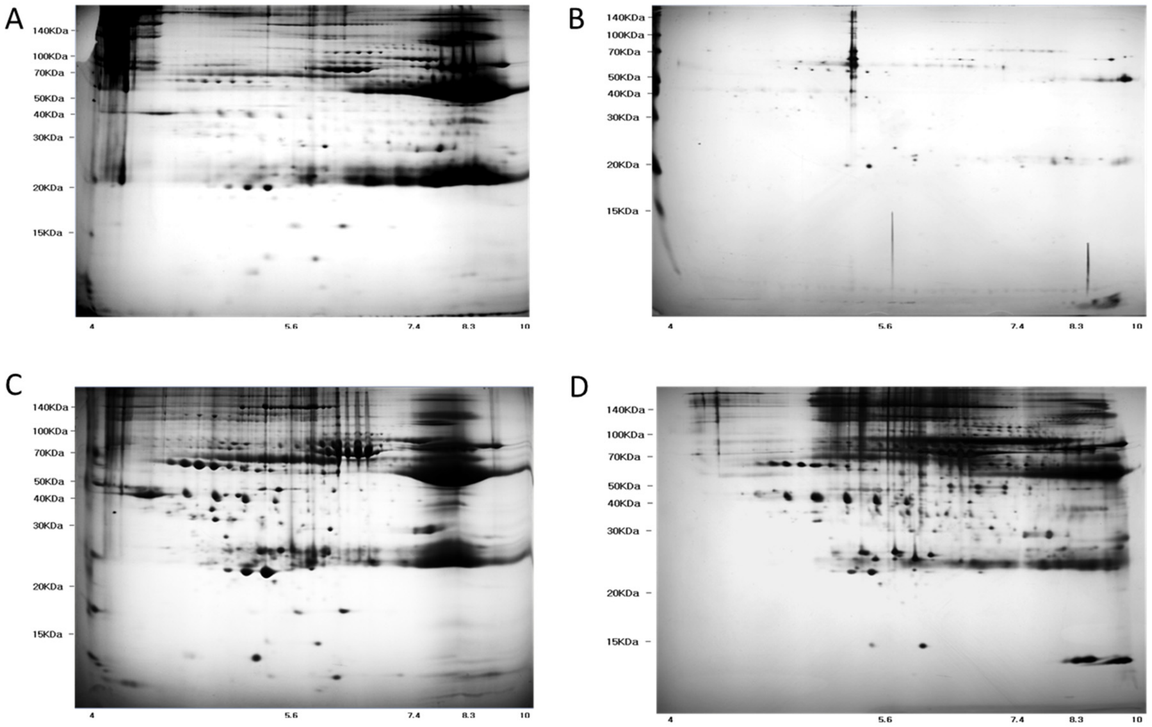

Figure S2. Images for 2-DE of EVs isolated by (**A**) PEG precipitation, (**B**) PEG precipitation and magnetic bead-based isolation, (**C**) SBI exosome isolation kit, and (**D**) Invitrogen exosome isolation kit.

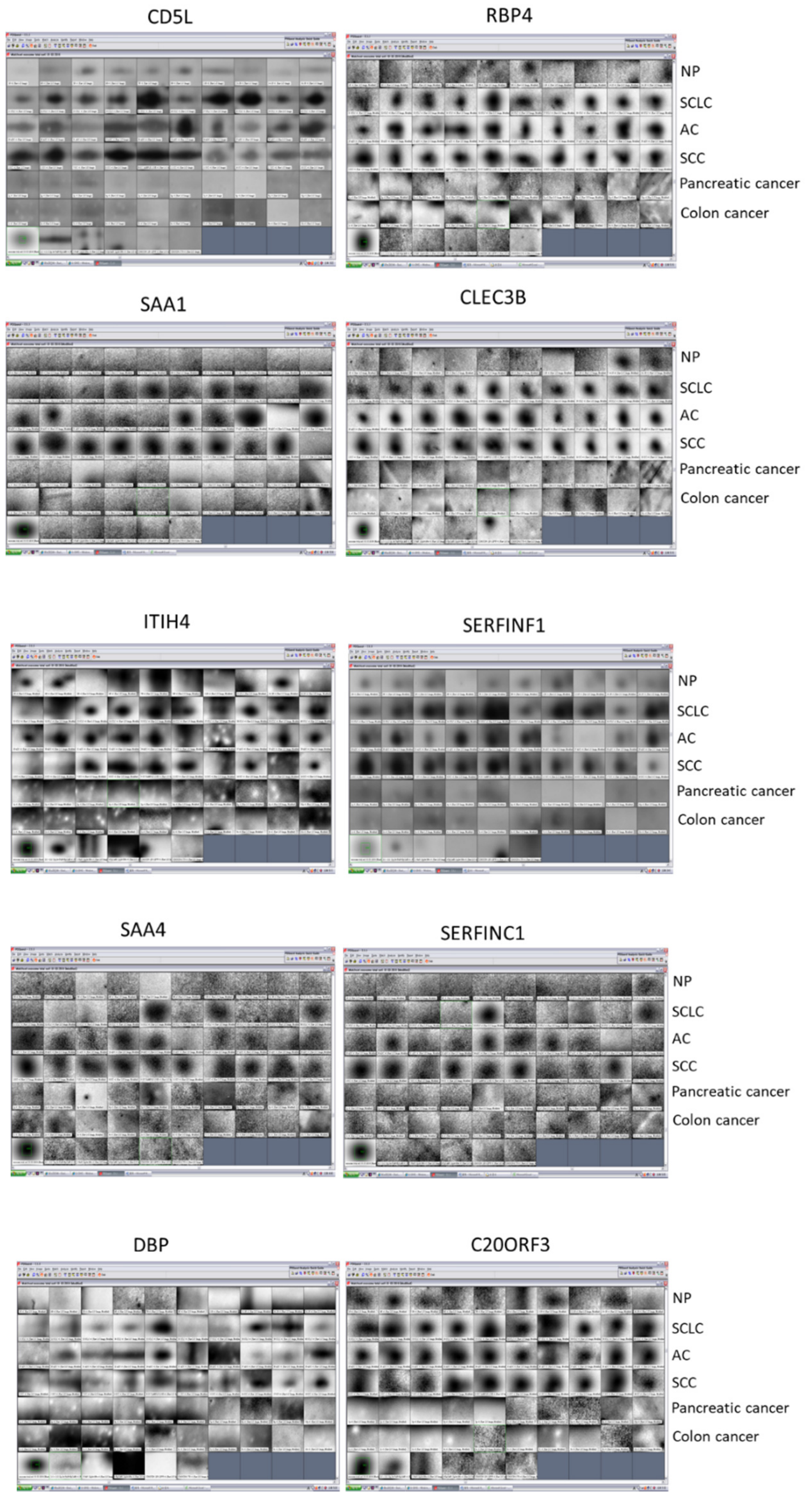

Figure S3. Zoomed protein spots in 2-D gel electrophoresis following Coomassie Brilliant staining, using serum-originated exosomes. Proteins were identified by MALDI-TOF/MS after excision of the gels. NP, normal person; SCLC, small cell lung cancer; AC, adenocarcinoma ling cancer; SCC, squamous carcinoma cancer.
